# Tea Bioactive Modulate Innate Immunity: In Perception to COVID-19 Pandemic

**DOI:** 10.3389/fimmu.2020.590716

**Published:** 2020-10-28

**Authors:** Pritom Chowdhury, Anoop Kumar Barooah

**Affiliations:** ^1^Department of Biotechnology, Tocklai Tea Research Institute, Tea Research Association, Jorhat, India; ^2^Directorate, Tocklai Tea Research Institute, Tea Research Association, Jorhat, India

**Keywords:** coronavirus, cytokines, innate immunity, micronutrients, nutritional immunity, tea polyphenols, vitamins

## Abstract

Innate immunity impairment led to disruption in cascade of signaling pathways upregulating pro-inflammatory cytokines, diminish interferons, depleted natural killer cells and activate reactive oxygen species production. These conditions severely affected body’s ability to fight against infectious diseases and also plays a pivotal role in disease progression. Here, in emphasis is on nutritional immunity for regulating effective innate immune response for combating against infectious diseases like novel coronavirus disease (COVID 19). Drawing from discoveries on in-vitro experiments, animal models and human trials, tea polyphenols, micronutrients, and vitamins has the potential to modulate and enhance innate immune response. This article provides a comprehensive review on tea (*Camellia sinensis* L*)* infusion (a hot water extract of dried processed tea leaves prepared from young shoots of tea plant) as an innate immunity modulator. Tea infusion is rich in polyphenols; epigallocatechin gallate (EGCG) and theaflavin (TF), major green and black tea polyphenols, respectively. Studies showed their immunomodulatory competence. Tea infusions are also rich in alkaloids; caffeine and its intermediates, theophylline and theobromine, which have anti-inflammatory properties. Tea plant being an acidophilic perennial crop can accumulate different micronutrients, *viz*., copper (Cu), iron (Fe), manganese (Mn), selenium (Se), and zinc (Zn) from growing medium, i.e., from soil, which led to their considerable presence in tea infusion. Micronutrients are integral part of innate immune response. Overall, this review presents tea infusion as an important source of nutritional immunity which can enhance innate immune response in order to mitigate the unprecedented COVID-19 pandemic.

## Introduction

Coronaviruses belongs to a group of enveloped, non-segmented positive-sense RNA viruses belongs to the *β* genus, *Nidovirales* order of the *Coronaviridae* family (1). A novel coronavirus, since named as severe acute respiratory syndrome coronavirus 2 (SARS-CoV-2) emerged in 2019, when it was first reported from Wuhan city of China causing respiratory illness (2). Soon it spread worldwide with cases reported from 182 countries and 33 territories and the World Health Organization has named it as coronavirus disease (COVID-19). Total reported cases surge past 26 millions among which more than 0.88 million succumbed to infection, which accounts for almost 3.26% mortality as on 07 September 2020 (3). On 11 March 2020, WHO declared COVID-19 as pandemic after almost 3 months from its first report (4). Information on COVID-19 pathophysiology is still emerging, but lots have been known till date, particularly on the role of immune response in disease progression (5, 6). Understanding immune regulation triggered by SARS-CoV-2 holds the key for development of vaccines and therapeutics regimen, which are in developing stages. Low neutralizing antibodies reported in a study, which is around 30% of COVID-19 recovered patients is a matter of concern (7). Variation in neutralizing antibodies response and antibody dependent enhancement should be a matter of investigation for effective vaccine candidates (7, 8). Earlier studies on SARS-CoV patients and present evidence on COVID-19 suggested that exacerbate host immune response rather than virulence of the infected virus is responsible for pulmonary injury and multiple organ dysfunctions (9, 10). Therefore, an effective therapeutic needs to address both anti-viral action as well as specific cytokine targeted immunomodulation. This complexity of COVID-19 warrants “multiple approaches” to combat disease progression. In addition to vaccines and effective drugs, a pivotal approach would be of nutritional immunity. This will be important not only to patients with underlying conditions like diabetes, hypertension, etc., but also for other infected and healthy population. Natural compounds in plants are our regular source of nutritious supplements. Among many sources, the tea plant (*Camellia sinensis*) being rich in antioxidant compound polyphenols, micronutrients, and vitamins in tea infusion makes it as an unique beverage. Tea being a popular and most consumed manufactured drink in the world can play an important role in nutritional immunity (11). The tea consumption worldwide stands at around 4.84 million tons (11). Furthermore, consumption of tea is popular thought out the world due to its easy availability as well as its economic viability. In this review, we have highlighted the importance of tea constituents on regulating an effective innate immune response in special perspective to present unprecedented pandemic. The Innate immunity is the first line of body’s defense against intruded pathogen and will be most important to constraint the pandemic spread in terms of role of nutritional immunity.

## Tea Polyphenols and Alkaloids Role in Innate Immunity

Immunological responses reported till now suggested a “cytokine storm” mediated disease severity (12, 13). Earlier studies on SARS-CoV and MERS-CoV, showed down regulation of type I Interferon (IFN), as a strategy to evade immune response (9). Present virus SARS-CoV-2 share close genomic proximity with SARS-CoV and MERS-CoV and it is most likely that SARS-CoV-2 has similar mechanism to evade innate immune response (14). Recent findings confirmed that critical COVID-19 patients have an impaired type I IFN production and a lower viral clearance (10). Concurrently, down regulation of interferon stimulated genes and also plasma levels of IFN- α2 protein in critical patient was observed whereas IFN-ß was undetectable in the mild, moderate, and critical categories of patients studied (10). It is noteworthy that patients with underlying disease conditions were excluded from the study. Studies showed that green tea polyphenol (−)epigallocatechin-3-gallate (EGCG) can induced IFN-λ 1, antiviral interferon stimulated genes expression in both Hepatitis C Virus (HCV), Japanese fulminant hepatitis (JFH) -1-infected and uninfected human hepatoma (Huh7) cells (15). The HCV is an RNA virus similar to SARS-CoV-2 and share same mode of action in reference to impairing interferons through down regulation of receptors like toll-like receptors (TLRs) and retinoic acid-inducible gene I (RIG-I) (16). Earlier studies on SARS-CoV reported the role of TLR3 adapter protein, Toll/interleukin-1 receptor domain-containing adapter-inducing interferon-β (TRIF) and TLR4 related TRIF-related adaptor molecule (TRAM) signaling in controlling viral loads in mice alveolar tissues (17). This protective signaling *via* TLR3/TRIF and TLR4/TRAM is postulated to be unique in the pathophysiology of coronaviruses unlike in influenza where their depletion does not change the pathogenesis (17). The same should be looked into SARS-CoV-2 interrogating their role both in clinical and experimental aspect. These may lead to importance of TLR agonist in controlling disease progression. Experimental studies with murine derived macrophage and dendritic cells showed that EGCG intervene TLR4 and TLR2 expression through down streaming mitogen-activated protein kinase (MAPK) and nuclear factor kappa B (NF-Kb) signaling leading to inhibition of pro-inflammatory cytokines (18, 19). Highly expressed TLR2 in alveolar epithelial lung tissue is another target which should be investigated (20). EGCG and theaflavin 3,3′ digallate (TFDG) were found to be a potent RIG-I inhibitors (21). Individuals at high risk to SARS-CoV-2 are those with underlying diseases, including diabetes, hypertension, and cardiovascular disease (14). Children are at lower risk which may be due to their adequate innate immune response. These facts strongly indicate that innate immune response is a critical factor for disease outcome. Mounting evidence suggested that polyphenols and micronutrients improve defense function, i.e., resistance to infection, by modulating immune regulation; this may have a strong implication in controlling COVID-19 particularly for cytokine storms (22) EGCG has inhibitory effect on neutrophil transmigration through monolayers of endothelial cells which in turn can reduce vascular permeability (23). It can also reduce neutrophil elastase, a proteolytic molecule implicated with increase permeability in alveolar epithelium (24). Loss of endothelial cells barrier integrity and production of reactive oxygen species by neutrophils leads to homeostatic imbalance which may explain the reason for acute respiratory distress syndrome as observed in COVID-19 patients. This can be characterized by alveolar tissue injury by inflammatory cytokines due to vascular leakage. The inhibitory action of EGCG and theaflavin (TF) against ROS and neutrophil elastase at a concentration available in blood (**Table 1**) is quite encouraging (23, 31, 32). EGCG and TF, a major black tea polyphenol suppresses the lipopolysaccharide (LPS)-induced intercellular adhesion molecule (ICAM)-1 and vascular cell adhesion molecule (VCAM)-1 expression through blockage of nuclear factor-kappa B (NF-κB) and c-Jun N-terminal kinase (JNK) activation pathway (33, 34). These findings probably explain the inhibitory mechanism of neutrophils by tea polyphenols as ICAM-1 and VCAM-1 are expressed on endothelial cells. Evidence of the stimulatory activity of tea polyphenols of different origins on natural killer (NK) cells with a biphasic effect was reported (35, 36). Treatment with polyphenols in mice model with upper airway inflammation showed increase in NK cells degranulation (37). Although further investigation will be required for elucidating the role of tea polyphenols on NK cells, it may be assumed that a similar action will allow us to highlight the pivotal implication it may have on COVID-19 patients. The NK cells were reported to deplete in COVID-19 patients marking the weakening immune response at the early stage of SARS-CoV-2 infection (37, 38). The NK cells depletion was inversely correlated with increase in expression of Natural Killer Cell Receptors (NKG2A) (39). The same has been observed in COVID-19 patients where NKG2A expression was up-regulated (40). Therefore, the available evidences suggest that the tea polyphenols down regulating NKG2A to induce NK cells can play an important role in early stages of virus infection. Another important aspect is “gut-lung” crosstalk, in recent times this phenomenon has gained lot of attention and it is widely recognized that an ideal gut microenvironment is essential for a balanced immune response not only for gastrointestinal track but also for maintaining respiratory homeostasis (41). Experimental and clinical evidences have indicated that host stress response activates macrophages and neutrophils in alveolar cells, leading to increase in pro-inflammatory cytokines and catecholamines (42). This signal is strongly correlated with growth and virulence of bacterial community like streptococcus pneumonia, klebsiella pneumonia perpetuating alveolar inflammation (42). This change in the inflamed lungs also leads to “gut dysbiosis” altering gut microbiota. This may explain severity of gastrointestinal problem, e.g., diarrhea in COVID 19 patients (41). The “gut-lung” axis is a bidirectional phenomenon, which can explain that an altered bacterial diversity in gut also can trigger a cascade of inflammatory signals in the lungs through butyrate, propionate and secondary bile acids secreted by bacteroides which can bind to receptors of dendritic cells and macrophages (41, 42). Murine and clinical intervention studies indicated that tea catechins have prebiotic activities which help in improving gut barrier integrity, decrease LPS containing gram negative bacterial community in the gut, and regulate intestinal tight junction protein expressions (42). Tea also contain alkaloids methylxanthine, namely, caffeine (CAF) and its intermediates, theophylline (TPI), and theobromine (TBR) (43). They have structural with purine nucleosides, which enable them to act as competitive inhibitor of adenosine receptor (43). This G-protein coupled receptor is widely present in immune cells in the form of different subtypes and plays a pivotal role in regulating innate immune response (43). These interactions of methylxanthines with adenosine receptor may be attributed to functional role of CAF in suppression of neutrophil, monocyte chemotaxis and inhibition of TNF-alpha in human blood (44). TPI is a proven vasodilator and used in the treatment of breathing condition (Apnea of prematurity) of neonates (43). Due to its anti-inflammatory action on bronchial airways, it may prove beneficial to treat ARDS patients when treated for shorter duration. TBR too has similar activity but is a weaker diuretic and muscle relaxant in comparison to TPI (45). Methylxanthine has also the capability to cross the blood brain barrier and regulate gamma-aminobutyric acid (GABA), a principle neurotransmitter in CNS (43). Studies in mice model showed GABA can control inflammatory cytokines in peripheral macrophages (46). This may have a positive implication in neuroprotection of COVID-19 patients with CNS dysfunction. Studies are warranted to explore this dimension with methylxanthines.

**Table 1:** Immune activities, content and recovery from human plasma of major green tea polyphenol, epigallocatechin gallate (EGCG), and black tea polyphenol, theaflavin (TF).

Tea Bioactives	Content in tea infusion (mg g^−1^)	Immune activities	Concentration in plasma (peak time)	References
Polyphenols				
EGCG	113.6 ± 5.85^a^	Enhances interferon secretion; enhances NK cell activity; regulates NF-κB, RIG-I dependent signal transduction; Inhibits ROS activity; regulates apoptosis of neutrophils; regulates Th1/Th2 polarization	55 nM (2 h)^b^ 37 nM^♂^–42 nM^♀^ (2 h)^c^	(25) (26) (27)
TF	0.70 ± 0.08^a^	Down regulate NF-κB,ICAM-1,VCAM-1; inhibits pro-inflammatory cytokines, e.g., IL-6, regulates neutrophils; inhibits ROS activity	8 nM^♂^–16 nM^♀^ (45 min)^d^	(28)
Alkaloids				
CAF	Green tea (GT):34.92 ± 0.93^a^; Black tea (BT):35.13 ± 3.02^a^	Regulates neutrophils; suppress monocyte chemotaxis; inhibits TNF-α; neuroprotective effect	30.89 µM (60 min)^e^	(29, 30)
TBR	GT:0.16 ± 0.03^a^ BT:0.38 ± 0.10^a^	Vasodilator; muscle relaxant	23.17 µM (120 min)^e^	
TPI	GT:1.54 ± 0.02^a^ BT:1.61 ± 0.05^a^	Vasodilator; anti-inflammatory in bronchial airways	23.17 µM (120 min)^e^	

a Tea infusion was prepared with 1 gram of green or black tea in 100 ml of boiled distilled water.

b Subject followed overnight fasting before taking 500 ml of green tea. Blood samples were collected at 0, 0.5, 1, 2, 4, 6, 8, and 24-h time interval in reference to drinking tea.

c Subjects were given 300 mg of EGCG (Teavigo^®^ capsules, 94% EGCG) with 100 ml of water. Blood sample was collected before the intervention and at 30, 60, 90, 120, 180, 240, and 360 min after the ingestion. The European Food Safety Agency (EFSA) based on clinical trial has recommended intake of EGCG/day to less than 800 mg.

d Volunteers consumed 700 mg TF mixed in 150 ml hot water. Symbol designation: ♂, male; ♀, women volunteers.

e Subjects followed 4 h fasting before taking 270 mg of caffeine (CAF) and 250 mg of each theobromine (TBR) and theophylline (TPI). Blood sample was collected before the intervention and at 0.25, 0.5, 0.75, 1, 1.5, 2, 3, 4, till 10 h after methylxanthine dose.

## Micronutrients and Vitamins in Innate Immunity

Micronutrients and vitamins are important component in the diet for an effective innate immune response (47). Although their status in COVID 19 patients is not yet known but earlier studies documented that selenium (Se), zinc (Zn), and vitamin B6 are associated with adverse clinical outcome in patients of infectious diseases (48). While the micronutrients and vitamin deficiency will lead to overall weakening of the innate immune response, the individual elements does play an important role in balancing immunity (47). Tea is also a source of these micronutrients and vitamins, but their contents; particularly micronutrients may vary with its geographical origin (48, 49). Here, we consider seven essential micronutrients like copper (Cu), iron (Fe), manganese (Mn), selenium (Se), zinc (Zn), and vitamins (B_2_, B_12_) which are present in tea infusion (**Table 2**). This will reflect the uptake of elements in the gastrointestinal tract constituting epithelial cells to predict the effect of habitual tea drinking on innate immunity. Se supplementation has been reported to increase the lymphocyte proliferation, NK cell activity and IFN production in human subjects (54). Se deficiency has been related with severe lung pathology in mice infected with influenza virus (55). It was postulated that Se deficiency has a role in increased viral mutation resulting in more virulent phenotype (56). Recent study from China documented association between higher death rates observed in COVID-19 patients from regions known to be Se deficient in population to that of other regions (57). This observation strongly supported earlier studies findings, where Se deficiency leads to higher pathogenicity in RNA viruses (55, 56). But this also highlights the need for further research on Se status in COVID-19 patients taking into account various confounding factors. Zn is essential for maintaining homeostasis in respiratory alveolar epithelium cells due to its antioxidant and anti-inflammatory activity (58). In vitro studies showed the inhibitory activity of Zn ionophore pyrithione against replication of SARS-coronavirus by effecting enzyme RNA polymerase (RNA dependent RNA polymerase, RdRp) (59). Zn deficiency in lungs, in relation to zinc-binding protein metallothionein (MT), mediates zinc partitioning from lung to liver during hyperoxia, which resulted in lung injury (60). In sepsis condition, Zn deficiency resulted in increased NF-κB p65 mRNA expression resulting in upregulation of target genes interleukin (IL)-1β, TNFα, and ICAM-1 and increase in proinflammatory cytokines IL-6, IL-8, and TNF (61). These cytokines are reported to induce cytokine storm in COVID patients. Zn supplementation reduced neutrophil infiltration and myeloperoxidase mediated oxidative damage which mediates protection in airway inflammation (61). Extensive macrophage infiltration was also reported from post-mortem lungs and kidneys of COVID-19 patients (62, 63). It is established in nutritional immunology that Zn homeostasis is crucial for proper functioning of macrophages (64). Macrophages also play an essential role in iron homeostasis, by recycling iron through phagocytosis of senescent red blood cells for hemoglobin production (65). Vice versa Fe has an effect on regulating macrophages in reducing NF-κB p65 translocation into the nucleus which leads to inhibition of pro-inflammatory cytokine expression (66). Cu too is essential micronutrient acting as co-factor for enzymatic reaction and signal transduction (66). It found itself in a razor edge between essentiality and toxicity in regards to its accumulation in cells beyond its requirement. However, concept in innate immunity is emerging that immune cells uses this toxic level of Cu to attack invading pathogens (67). Activated macrophages during infection transport Cu to phagosome through high affinity copper uptake protein 1 and ATPase Copper Transporting Alpha (67). Cu deficiency has been related with impaired neutrophil and macrophage function (67). Recent literature supported a potential treatment using Cu as ant-viral and inflammatory along with N-acetylcysteine (NAC), colchicine, Nitric oxide and an effective anti-viral drug (68). Mn supplementation was found to increase TYPE I IFN production in murine models and in THP-1, a human macrophage cell line (69). Studies in mice model observed relation between Mn deficiency and impaired anti-viral response. Mn^2+^ enhances activity of PAMP molecule for binding to cyclic-GMP-AMP synthase (cGAS). Overall it helps in mediating stimulation of cGAS- STING (stimulator of interferon genes) signaling pathway for interferon release (69). Vitamins also has role in inhibition of inflammatory cytokines (70). Tea infusion is also an important source of vitamin B_12_ (53, 71). Studies showed that drinking tea can improve considerably B_12_ status in B_12_-deficient rats (72). It is an essential for innate immunity and its deficiency resulted in reduced white blood cells resulting in increased susceptibility to infections. Tea infusion is also source of another important vitamin, riboflavin (vitamin B_2_) (52). Its deficiency is a matter of concern for both developing and developed nations. Elderly, pregnant women and alcohol abusers are usually at high risk of immune suppression associated with its deficiency. It affects balanced macrophage functioning which results in reduction of cell viability and excess release of TNF-α (73). It is also known to down-regulate the NF-κB activation initiated by ROS (73), which are the potent activators of a plethora of general pro-inflammatory cytokines such as IL-6 and TNF-α; the two most prominent in “cytokine storm” in COVID-19 patients (5).

**Table 2:** Immune activities and content of micronutrients and vitamins in tea infusion.

Micronutrients and vitamins	Immune activities	Content in tea infusion	Actual body requirement	Reference
Selenium	Acts as immunostimulatory; helps in regulating oxidative stress; regulates phagocytic activity of macrophages; enhances T-cell proliferation, NK-cell mediated cytotoxicity; increases NK cell, lymphocytes, and leukocyte counts; modulates antioxidant capacity of lungs	Se-rich green tea (GT) - 1.44 μg Se/g DW; Regular GT - 0.13 μg Se/g DW; Se-rich black tea (BT) - 1.42 μg Se/g DW; Regular-BT - 0.08 μg Se/g DW	55 ug/day	(50)
Iron	Promotes activation of NF-kB cells; enhances host resistance to intracellular pathogens; regulates anti-microbial activity of macrophages	GT: 0.142–0.099 mg/L BT: 0.100–0.084 mg/L	12–15 mg/day	(51)
Zinc	Enhances phagocyte activities and cytokine secretion of macrophages; acts as antioxidant against reactive oxygen and nitrogen species; important for development and function of neutrophils and NK cells; Zn deficiency leads to decreased lymphocyte counts	GT: 0.130–0.588 mg/L BT: 0.113–0.057 mg/L	10–15 mg/day	(51)
Copper	Target invading pathogens in the phagosome with activated macrophages. Neutrophil and monocytes activities impaired with deficiency in Cu. Enhances NK cells	GT: 0.033–0.191 mg/L; BT: 0.072–0.441 mg/L	900 ug/day	(51)
Manganese	Stimulation of interferon production	GT: 0.78–3.94 mg/L; BT: 0.56–7.49 mg/L	1.8–2.3 mg/day	(51)
Vitamin B_2_	Essential for proper functioning of macrophages; B2 deficiency leads to decreases pathogen recognition and impaired immune response	GT: 3.26–2.80 µg/gm BT: 3.34–0.58 µg/g;	1.8–2.5 mg/day	(52)
Vitamin B_12_	Acts as immunomodulator; enhances NK cell activity; increases lymphocyte count; enhances T-cell proliferation	GT: 0.046–0.263 µg/g DW BT: 0.104–0.859 µg/g DW;	2.4 ug/day	(53)

Selenium enriched tea plantation is being practiced particularly in regions with low soil Se content and human Se deficient areas, as part of Se-biofortification. Infusions were prepared with 1 g of each tea incubated for 5 min in 50–100 ml of Milli-Q (MQ), or boiled distilled water.

## Discussion

**Figure 1:**
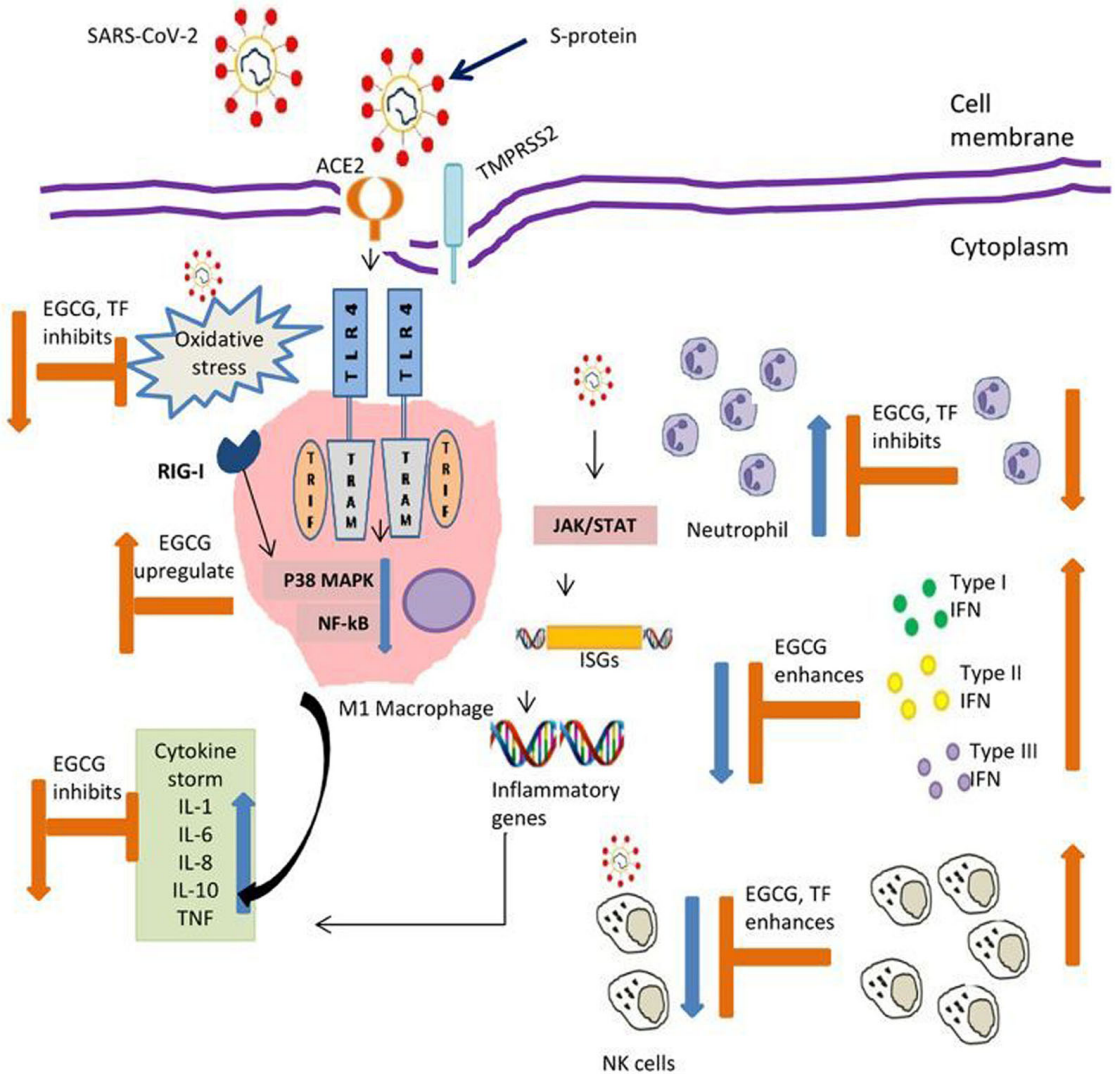
Possible action by tea polyphenols contributing to balance innate immunity in COVID-19. EGCG, Epigallocatechin gallate; TF, Theaflavins; ACE2-Angiotensin, converting enzyme 2; TMPRSS2 Transmembrane protease, serine 2; S-protein, Spike protein; TLR-4, Toll like receptor 4; TRIF, Toll/interleukin-1 receptor (TIR)-domain-containing adapter-inducing interferon-β; TRAM, TRIF-related adaptor molecule; RIG-1, Retinoic acid-inducible gene I; MAPK, Mitogen-activated protein kinases; NF-Kb, Nuclear factor kappa B; JAK, Janus kinase; STAT, Signal transducer and activator of transcription; ISGs, Interferon stimulating genes; IL- Interleukin; TNF, Tumor necrosis factor; IFN, Interferon; NK, Natural killer cells. Symbol denotes: Blue arrow reflects effect of virus; Orange arrow reflect action of polyphenols.

Innate immunity is pivotal as the first line of defense against viral infections. Ability of an infectious agent to evade innate immune response is determinant for its pathogenecity, augmenting severe symptoms or even fatality. In critical patients infected with SARS-CoV-2; respiratory failure, systemic shock and multi organ failure are common characteristics (74). Almost 30% of COVID 19 patients require intensive care unit for ventilation support (74). Acute respiratory distress syndrome is a common complication for viral pneumonia (74), which is same for SARS-CoV-2, SERS-CoV, and MERS augmented pneumonia cases. Pulmonary and alveolar tissue specific inflammatory responses are result of innate immune response leading to cascade of inflammatory host signaling pathways (38). The cross talk between innate and adaptive immunity is hallmark for immune strategies to combat disease progression. NK cells are one such example of crosstalk, which can recognize antigen and generate antigen dependent memory (75). Although understanding the immune response in COVID-19 patients is in growing stage, observations reported till now showed apparent involvement of hyper-inflammation and vascular permeability contributing towards the disease severity (12, 13, 38). Mounting evidence suggested that micronutrients improve defense function, i.e., resistance to infection, by modulating immune regulation (48). This include inhibition of pro-inflammatory cytokines, promotion of anti-inflammatory functions, modulation of cell-mediated immunity, alteration of antigen-presenting cell functions, and communication between the innate and adaptive immune systems. Evidence presented here suggested that tea constituting free radical scavengers polyphenols, micronutrients and vitamins has the functional ability to regulate all these functions. Numerous research work in recent times on TFs and EGCG, showed their importance as immunomodulator (**Figure 1**). Several medicinal plants highlighted for their medicinal properties in connection with human health in recent past. However, tea itself as a non-alcoholic beverage did not get much attention as a source of nutritional supplements. Being the most widely consumed drink globally, it is of paramount importance to highlight and harness the benefits of tea drinking. There are numerous different types of tea like black tea, green tea, oolong tea, white tea, yellow tea, etc., which differ due to difference in processing methods but have originated from the same plant *Camellia sinensis* L. Different tea has different major constituents like TFs in black tea and EGCG for green tea. Sometimes number of constituents in the same type of tea varies as per the geographical origin. Assam variety cultivars have been found to possess more polyphenol content than to China or Cambodia variety (76). Likewise, Darjeeling tea is rich in aroma which can be attributed to rich volatile compounds (77). These compounds may have beneficial effect on our respiratory system. Similarly, teas of other region have some unique characteristics, may be in terms of micronutrient contents. These all make tea an important component for nutritional research. Interestingly, computational modeling studies showed the potency of theaflavin-3,3’-digallate, a major TFs, and EGCG in inhibiting RNA-dependent RNA polymerase (RdRp) and the main protease (M^pro^), respectively. Both are key components of coronavirus replications and can be major drug target for SARS-CoV-2 (78–80). Earlier studies on HCV showed ability of TFs acting directly against virions particle, changing its morphology, making it unable to enter cells (81). The absorption of polyphenols involves small intestine, liver, and epithelial colon cells of large intestine through process of methylation, glucuronidation, and sulfation (82). They are too hydrophilic to penetrate the gut wall by passive diffusion and mainly active transport mechanism is involved in its absorption (82). Studies in mice model and human volunteers showed their presence in numerous tissues (83, 84). They are also found to be associated with better pulmonary functions in epidemiological studies (85). These findings are encouraging to consider polyphenols as an integral part of nutritional immunity particularly innate immune response, and tea being widely consumed drink is an important and unique source of essential micronutrients and vitamins as well.

## Conclusion

It is evident that innate immunity plays an important role in COVID-19 disease progression. This review showed the importance of nutritional immunity on innate immune response and tea as an important source of it. Recent advancements on increasing the bioavailability of tea polyphenols and its popularity as a beverage advocates for random control clinical trials to translate the basic research findings into clinical use. Nevertheless, this article will further boost tea as a health drink and give an add-on to nutritional supplements augmenting innate immunity in controlling the present pandemic. In present scenario, it will be interesting to study the effect of tea polyphenols on the respiratory and alveolar human cell lines regulating the inflammatory markers by mitigating the cytokine storm of SARS-CoV-2 infection. This will further justify the need for in-vivo studies and clinical trials for developing it as a nutraceuticals and popular health drink at large.

## Author Contributions

PC conceptualized, performed literature review, wrote, and presented (table and figures) the MS. AB conceptualized and helped in critically reviewing the MS. All authors contributed to the article and approved the submitted version.

## Conflict of Interest

The authors declare that the research was conducted in the absence of any commercial or financial relationships that could be construed as a potential conflict of interest.

## Abbreviations

IFN, Interferon; NK, Natural killer cells; IL, Interleukin; EGCG, Epigallocatechin-3-gallate; TF, Theaflavin; LPS, Lipopolysaccharide; NF-Kb, Nuclear factor kappa B; MAPK, Mitogen-activated protein kinase; Mpro, Main protease; RdRp, RNA-dependent RNA polymerase; RIG-I, Retinoic acid-inducible gene I; TLR, Toll like receptor; ICAM-1, Intercellular adhesion molecule; VCAM, Vascular cell adhesion molecule.
